# Usability and Acceptability of Technologies for Remote Home-based Environmental Monitoring in Older Adults

**DOI:** 10.1093/geroni/igaf122.2813

**Published:** 2025-12-31

**Authors:** Peyton Berning, Pei-An Lee, Wanting Yu, Brad Manor, Lewis Lipsitz, Amir Baniassadi

**Affiliations:** Hebrew SeniorLife, Boston, Massachusetts, United States; Hebrew SeniorLife & Harvard Medical School, Boston, Massachusetts, United States; Hebrew SeniorLife, Boston, Massachusetts, United States; Hebrew SeniorLife/Harvard Medical School, Boston, Massachusetts, United States; Hebrew Senior Life, Boston, Massachusetts, United States; Hebrew SeniorLife, Boston, Massachusetts, United States

## Abstract

Wearable and smart-home technologies offer exciting opportunities to study environmental effects on health directly within individuals’ homes. Yet, it is unclear whether older adults are capable of using these devices. This pilot study examined older adults’ receptiveness and ability to use smart thermostats and watches to monitor sleep under different bedroom temperatures. Fourteen independently-living, smartphone-owning older adults (age 82.1±5.6, 7 female, MoCA score: 24-30) were equipped with cloud-controlled thermostats and given watches requiring daily syncs across two apps. Participants received 30 minutes of at-home training supplemented with graphics and could contact study staff as needed. Eleven participants were equipped with both devices in one visit, while three required additional visits. Overall, we collected 569 person-nights of data while remotely adjusting bedroom temperature. No participants needed additional thermostat training. Participants adhered to wearing the watch 96% of the time. Ten watch-related troubleshooting calls were made, six of which concerned smartphone apps. A subset of eight participants answered questions about interacting with study technology. All eight found thermostat use “very easy,” as did six for watch and app use. Two found watch and app use “somewhat easy.” Four attributed successful technology usage to self-motivated routine building. Critiques included discomfort sleeping with watches and non-intuitive app interfaces. All found overall participation “very easy.” Older adults appear accepting and capable of using wearable and smart-home technologies with ongoing support, indicating untapped opportunities to study their environmental health. Future studies should explore wearables promoting usability and include populations with lower cognitive and technology experience levels.

